# Triglyceride‐Glucose‐Waist Circumference Index as a Surrogate Marker for Visceral Obesity: Analysis of NHANES 2011–2018

**DOI:** 10.1002/osp4.70163

**Published:** 2026-08-25

**Authors:** Pan Fu, Yue Li, Keqin Zhang

**Affiliations:** ^1^ Center of Urology and Nephrology The Second Affiliated Hospital of Chongqing Medical University Chongqing China

Excess visceral adipose tissue (VAT) is a metabolically adverse fat depot that has been strongly linked to cardiometabolic dysfunction, cardiovascular disease, several cancers, and premature mortality [1]. Direct imaging methods such as computed tomography, magnetic resonance imaging, and dual‐energy X‐ray absorptiometry (DXA) provide robust assessment of VAT, but their routine use in large‐scale screening and everyday clinical practice remains constrained by cost, access, and logistics [2, 3]. Therefore, simple surrogate markers that can approximate VAT using anthropometric and laboratory data remain clinically valuable.

Traditional anthropometric indices, particularly waist circumference (WC) and body mass index (BMI), have long been used as pragmatic surrogates of excess adiposity, and previous work suggests that WC and BMI are among the strongest simple markers of visceral adiposity [4]. However, anthropometry alone may not fully capture the metabolic component of unhealthy fat distribution. The triglyceride‐glucose (TyG) index is a practical surrogate of insulin resistance, and TyG‐related composite indices may better reflect the combined metabolic and central obesity burden that characterizes visceral obesity [5].

Recent studies have shown that TyG‐based indices are associated with visceral obesity in both diabetic and non‐diabetic settings. Specifically, the TyG index showed meaningful predictive value for visceral obesity and body fat distribution in patients with type 2 diabetes, while TyG and its combinations with anthropometric obesity indicators were also reported to predict DXA‐measured visceral obesity in a generally healthy population [6, 7]. Nevertheless, comparative data on TyG‐WC against established indices such as WC and BMI in a nationally representative US sample have remained limited, particularly with respect to sex‐specific thresholds and agreement with conventional anthropometric classification.

Adults aged 18 years or older who participated in NHANES 2011–2018 were included [2]. Participants without VAT data or without complete data for BMI, WC, fasting plasma glucose, triglycerides, and high‐density lipoprotein cholesterol were excluded. VAT measured by DXA was used as the reference standard. The surrogate markers evaluated were BMI, WC, TyG, TyG‐BMI, TyG‐WC, and visceral adiposity index (VAI). VAI was calculated using sex‐specific formulas. Pearson correlation analysis was used to assess the relationship between each marker and continuous VAT. High VAT was defined as the sex‐specific upper quartile of VAT. Receiver operating characteristic (ROC) analysis was used to evaluate diagnostic performance overall and by sex. Areas under the ROC curves (AUCs) were compared using DeLong's test. For each marker, the optimal cutoff was determined by maximizing the Youden index, as originally described by Youden [8]. Sensitivity, specificity, positive predictive value (PPV), negative predictive value (NPV), and accuracy were calculated. Agreement between TyG‐WC and established adiposity indices was assessed using Cohen's kappa.

A total of 5745 participants were included, comprising 2924 males and 2821 females, with a median age of 38 years (interquartile range [IQR], 27–49 years). High VAT, defined by the sex‐specific upper quartile of VAT, was present in 25.00% of the overall cohort. The median VAT was 90.83 cm^2^ (IQR, 55.08–134.33). Correlation analysis showed that TyG‐WC had the strongest correlation with continuous VAT overall (*r* = 0.80, *p* < 0.001) and in both males (*r* = 0.78, *p* < 0.001) and females (*r* = 0.82, *p* < 0.001), followed by WC, TyG‐BMI, BMI, TyG, and VAI (Table 1).

**TABLE 1:** Pearson correlations between surrogate markers and VAT overall and by sex.

Marker	Overall *r* (95% CI)	*p*	Male *r* (95% CI)	*p*	Female *r* (95% CI)	*p*
BMI	0.67 (0.66–0.69)	< 0.001	0.65 (0.62–0.67)	< 0.001	0.71 (0.69–0.73)	< 0.001
WC	0.77 (0.76–0.78)	< 0.001	0.75 (0.73–0.77)	< 0.001	0.78 (0.77–0.80)	< 0.001
TyG	0.50 (0.48–0.52)	< 0.001	0.47 (0.44–0.50)	< 0.001	0.53 (0.50–0.55)	< 0.001
TyGBMI	0.74 (0.73–0.75)	< 0.001	0.70 (0.68–0.72)	< 0.001	0.78 (0.76–0.79)	< 0.001
TyGWC	0.80 (0.79–0.81)	< 0.001	0.78 (0.77–0.79)	< 0.001	0.82 (0.81–0.83)	< 0.001
VAI	0.30 (0.28–0.33)	< 0.001	0.30 (0.26–0.33)	< 0.001	0.31 (0.27–0.34)	< 0.001

*Note:* Values represent Pearson correlation coefficients (*r*). All *p* values < 0.001.

TyG‐WC showed the best diagnostic performance for identifying high VAT overall (AUC = 0.90, 95% CI: 0.89–0.91), in males (AUC = 0.89, 95% CI: 0.88–0.90), and in females (AUC = 0.91, 95% CI: 0.90–0.92) (Table 2; Figure 1). The corresponding optimal cutoffs were 860.76 overall, 903.79 in males, and 860.70 in females. WC ranked second overall and in males, with AUCs of 0.88 and 0.87, respectively, whereas TyG‐BMI and WC showed similar performance in females, with both AUCs rounded to 0.89, and the difference was not statistically significant (DeLong's test, *p* = 0.33). BMI showed lower discrimination (AUCs, 0.84 overall, 0.82 in males, and 0.86 in females). DeLong's test further showed that TyG‐WC significantly outperformed WC, BMI, TyG, TyG‐BMI, and VAI in the overall cohort and in both sexes (all *p* < 0.001).

**TABLE 2:** Diagnostic performance of surrogate markers for identifying high VAT overall and by sex.

Group	Marker	AUC (95% CI)	Cutoff	Sensitivity	Specificity	PPV	NPV	Accuracy
Overall	BMI	0.84 (0.83–0.85)	28.45 kg/m²	84.50%	68.40%	47.10%	93.00%	72.40%
Overall	WC	0.88 (0.87–0.89)	98.65 cm	86.80%	73.60%	52.30%	94.40%	76.90%
Overall	TyG	0.76 (0.75–0.77)	8.49	76.70%	63.60%	41.20%	89.10%	66.90%
Overall	TyGBMI	0.87 (0.86–0.88)	245.22	88.20%	70.30%	49.70%	94.70%	74.80%
Overall	TyGWC	0.90 (0.89–0.91)	860.76	87.70%	76.60%	55.60%	94.90%	79.40%
Overall	VAI	0.76 (0.75–0.78)	3.22	73.80%	66.40%	42.30%	88.40%	68.30%
Male	BMI	0.82 (0.81–0.84)	28.45 kg/m²	80.20%	69.80%	47.00%	91.30%	72.40%
Male	WC	0.87 (0.86–0.89)	98.85 cm	87.50%	71.70%	50.80%	94.50%	75.70%
Male	TyG	0.75 (0.73–0.77)	8.72	70.00%	69.60%	43.50%	87.50%	69.70%
Male	TyGBMI	0.85 (0.84–0.87)	250.85	82.50%	73.20%	50.60%	92.60%	75.50%
Male	TyGWC	0.89 (0.88–0.90)	903.79	81.40%	81.30%	59.20%	92.90%	81.30%
Male	VAI	0.75 (0.73–0.76)	3.17	74.60%	64.40%	41.10%	88.40%	66.90%
Female	BMI	0.86 (0.84–0.87)	27.85 kg/m²	93.20%	63.70%	46.10%	96.60%	71.10%
Female	WC	0.89 (0.87–0.90)	97.05 cm	88.60%	73.20%	52.50%	95.10%	77.10%
Female	TyG	0.78 (0.76–0.80)	8.38	76.40%	66.50%	43.20%	89.50%	69.00%
Female	TyGBMI	0.89 (0.88–0.90)	245.22	91.20%	70.80%	50.90%	96.00%	75.90%
Female	TyGWC	0.91 (0.90–0.92)	860.70	85.00%	82.10%	61.30%	94.20%	82.80%
Female	VAI	0.78 (0.76–0.80)	2.84	81.40%	61.10%	41.10%	90.80%	66.20%

*Note:* High VAT was defined as the sex‐specific upper quartile of VAT. Optimal cutoffs were derived by maximizing the Youden index [8].

Abbreviations: AUC, area under the curve; CI, confidence interval; NPV, negative predictive value; PPV, positive predictive value.

**FIGURE 1:**
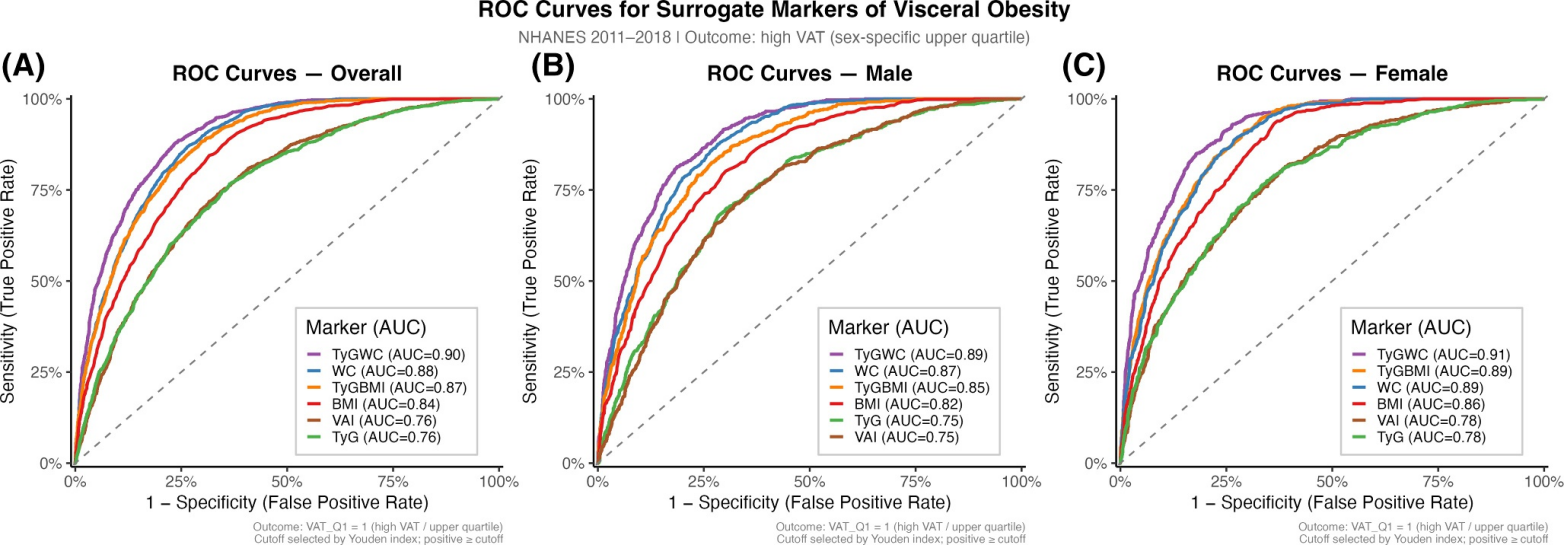
Receiver operating characteristic (ROC) curves of surrogate markers for identifying high VAT in the overall cohort (A), males (B), and females (C). BMI, body mass index; ROC, receiver operating characteristic; TyG, triglyceride‐glucose index; TyG‐BMI, triglyceride‐glucose body mass index; TyG‐WC, triglyceride‐glucose waist circumference index; VAI, visceral adiposity index; WC, waist circumference.

Using Youden‐derived thresholds, TyG‐WC also showed favorable threshold‐based performance. In the overall cohort, TyG‐WC achieved a sensitivity of 87.70%, specificity of 76.60%, PPV of 55.60%, NPV of 94.90%, and accuracy of 79.40%. In males, the TyG‐WC cutoff of 903.79 yielded a sensitivity of 81.40%, specificity of 81.30%, PPV of 59.20%, NPV of 92.90%, and accuracy of 81.30%. In females, the TyG‐WC cutoff of 860.70 yielded a sensitivity of 85.00%, specificity of 82.10%, PPV of 61.30%, NPV of 94.20%, and accuracy of 82.80%. Agreement analysis showed substantial concordance between TyG‐WC and WC (*κ* = 0.77 overall, *κ* = 0.70 in males, and *κ* = 0.76 in females) and moderate‐to‐substantial concordance between TyG‐WC and BMI (*κ* = 0.64 overall, *κ* = 0.64 in males, and *κ* = 0.61 in females) (Table 3). Additional agreement results for TyG‐WC, WC, and BMI against high VAT classification are provided in Supporting Information Table S1. These findings suggest that TyG‐WC remains closely aligned with established anthropometric indices, especially WC, while providing better discrimination.

**TABLE 3:** Agreement of TyGWC with WC and BMI using Youden‐derived thresholds.

Comparison	Overall *κ* (agreement %)	Male *κ* (agreement %)	Female *κ* (agreement %)
TyGWC versus WC	0.77 (88.80%)	0.69 (85.40%)	0.76 (88.60%)
TyGWC versus BMI	0.64 (82.60%)	0.64 (82.90%)	0.61 (80.30%)

*Note:* Agreement assessed using Cohen's kappa. Thresholds were derived by maximizing the Youden index [8]. Overall/Male/Female columns show *κ* (observed agreement %).

TyG‐WC may perform better because it reflects both central adiposity and metabolic disturbance, which may explain why it consistently outperformed WC, BMI, TyG, TyG‐BMI, and VAI. WC remained a strong and highly practical marker, and its performance in this study confirms why it has retained clinical value as a simple surrogate of abdominal adiposity [4]. However, the present findings suggest that when fasting triglyceride and glucose data are already available, TyG‐WC may provide incremental value over WC alone for identifying individuals with high VAT.

The sex‐specific thresholds observed here are also clinically relevant. TyG‐WC performed slightly better in females than in males, and the optimal cutoff was meaningfully lower in females. Prior work has shown that VAT may have sex‐specific associations with cardiometabolic and cardiovascular risk, supporting the view that a single universal threshold may not be appropriate for both sexes [9]. Accordingly, sex‐stratified cutoffs may be more appropriate when TyG‐WC is used for screening or risk enrichment.

Although the NHANES cycles used in this study are not recent, they remain highly informative because they provide one of the few publicly available, nationally representative US datasets that combine standardized DXA‐derived VAT measurements with fasting laboratory variables and anthropometric data. A recent study has continued to use these NHANES 2011–2018 DXA‐VAT data to examine obesity‐related disease risk, indicating that the dataset remains relevant for contemporary methodological and epidemiological questions [10]. At the same time, several limitations should be acknowledged. First, the study was cross‐sectional and therefore cannot establish longitudinal or causal relationships. Second, high VAT was defined using a sex‐specific upper‐quartile approach rather than an outcome‐based clinical threshold. Third, external validation in independent populations is still needed before the proposed cutoffs are generalized.

In conclusion, TyG‐WC showed the best overall performance as a surrogate marker of high VAT in US adults from NHANES 2011–2018. It outperformed established indices while maintaining substantial agreement with WC and moderate‐to‐substantial agreement with BMI. These findings support TyG‐WC as a practical screening‐oriented marker of visceral obesity, particularly when routine fasting laboratory data are already available.

## Funding

The authors have nothing to report.

## Conflicts of Interest

The authors declare no conflicts of interest.

## Supporting information


**Table S1:** Agreement of TyG‐WC, WC, and BMI with high VAT using Youden‐derived thresholds.

## Data Availability

The data that support the findings of this study are openly available in the National Health and Nutrition Examination Survey at https://www.cdc.gov/nchs/.
