# Supplementary material for: Triglyceride‐Glucose‐Waist Circumference Index as a Surrogate Marker for Visceral Obesity: Analysis of NHANES 2011–2018
Source: Obes Sci Pract. 2026 Aug 25;12(4):e70163. doi: 10.1002/osp4.70163 (PMC13505882; doi:10.1002/osp4.70163)
Supplement: Supplementary file 1 [file OSP4-12-e70163-s001.docx]

**Supplementary Table S1. Agreement of TyG-WC, WC, and BMI with high VAT using Youden-derived thresholds**

*High VAT was defined as the sex-specific upper quartile of VAT. Agreement assessed using Cohen's kappa. Thresholds were derived by maximizing the Youden index. ^8^ TP, true positive; TN, true negative; FP, false positive; FN, false negative.*

| **Marker** | **Group** | **κ** | **SE** | **Agreement %** | **TP** | **TN** | **FP** | **FN** | **n** |
| --- | --- | --- | --- | --- | --- | --- | --- | --- | --- |
| TyG-WC | Overall | 0.54 | 0.01 | 79.40% | 1259 | 3302 | 1007 | 177 | 5745 |
| TyG-WC | Male | 0.56 | 0.02 | 81.30% | 595 | 1783 | 410 | 136 | 2924 |
| TyG-WC | Female | 0.59 | 0.02 | 82.80% | 599 | 1738 | 378 | 106 | 2821 |
| WC | Overall | 0.50 | 0.01 | 76.90% | 1247 | 3171 | 1138 | 189 | 5745 |
| WC | Male | 0.48 | 0.02 | 75.70% | 640 | 1573 | 620 | 91 | 2924 |
| WC | Female | 0.50 | 0.02 | 77.10% | 625 | 1550 | 566 | 80 | 2821 |
| BMI | Overall | 0.42 | 0.01 | 72.40% | 1213 | 2946 | 1363 | 223 | 5745 |
| BMI | Male | 0.40 | 0.02 | 72.40% | 586 | 1531 | 662 | 145 | 2924 |
| BMI | Female | 0.42 | 0.01 | 71.10% | 657 | 1349 | 767 | 48 | 2821 |
